# Development of a radiopaque, long-term drug eluting bioresorbable stent for the femoral-iliac artery†

**DOI:** 10.1039/c9ra06179g

**Published:** 2019-10-28

**Authors:** Dong-Heon Ha, Jae Yun Kim, Tae Sik Park, Jong Ha Park, Suhun Chae, Byoung Soo Kim, Han Cheol Lee, Dong-Woo Cho

**Affiliations:** Department of Mechanical Engineering, Pohang University of Science and Technology Pohang 37673 Korea dwcho@postech.ac.kr; School of Interdisciplinary Bioscience and Bioengineering, Pohang University of Science and Technology Pohang 37673 Korea; Division of Cardiology, Department of Internal Medicine, College of Medicine, Pusan National University Busan 602-739 Korea

## Abstract

Tubular tissues exist in various forms purported for blood supply, waste secretion, *etc.* to aid proper function and maintenance of the human body. Under pathological conditions, however, these tissues may undergo stenosis. A major surgical treatment for stenosis is to implant a medical device called a stent which aims to expand the narrowed tissue and maintain its patency. Most stents are currently made from metals; despite their high mechanical strength, however, interactions with the host tissue often results in restenosis and stent fracture. To solve these problems, a bioresorbable stent (BRS) is proposed as a next generation stent. In this study, a rotating rod combined 3D printing system was developed to fabricate various types of BRSs. In addition, we confirmed that a 1.5 year long-term release of paclitaxel is possible using polymeric materials. Moreover, a stent loaded with contrast powder was fabricated and was successfully viewed under fluoroscopy. The stent was then implanted in the iliac arteries of pigs and no adverse events were observed for up to 8 weeks.

## Abstract 2

A radiopaque, long-term drug eluting bioresorbable stent is developed for the treatment of femoral-iliac artery. The prepared materials are printed on a pre-designed rotating rod. The fabricated stent can be adapted for various clinical cases.

## Graphical abstract

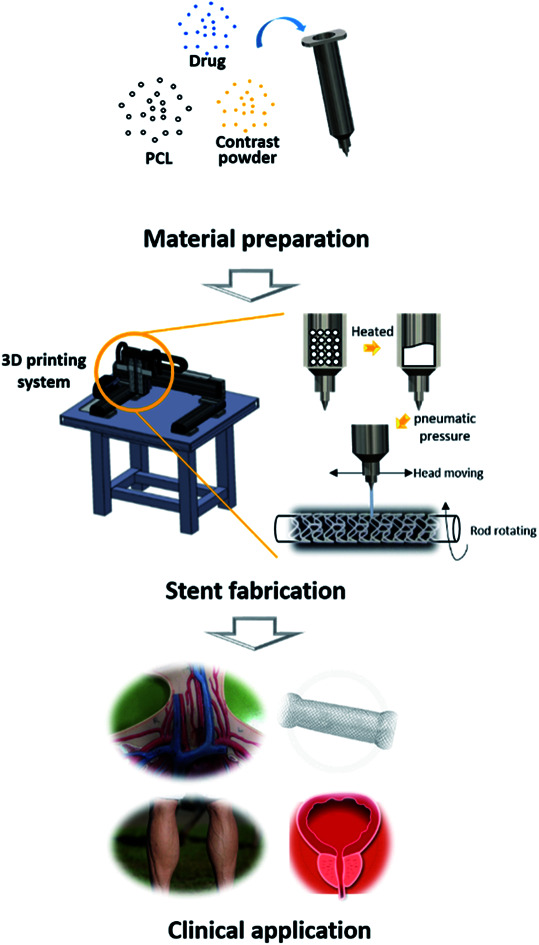

## Introduction

The human body contains multiple types of tubular tissues including blood vessels, the esophagus, trachea, bile duct, and urethra, and several causes – cancer, atherosclerosis, inflammation, infection, calcification, *etc.* – lead to stenosis of these tissues.^1,2^ Stenosis of these tubular tissues can result in leg pain and paraesthesia, difficulty in ingesting food, tissue necrosis, and even death.^1,3–6^ When such problems occur a stent is inserted so as to secure patency upon opening a clogged tissue.

Most stents are fabricated by machining metal tubes using laser cutting technology and remain in the body for a lifetime.^7^ The stents that are implanted undergo host tissue interaction, and may lead to various problems. For example, stents with a mechanical strength exceeding that of the host tissue can stimulate tissue restenosis,^8,9^ while the reverse may lead to fracture and breakage due to repeated force exerted on the stent from the surrounding tissue.^10^

In the case of stents used in the treatment of femoral-iliac artery, the patency rate is in the range of 40–70% for bare metal stents and 65–80% for drug eluting stents in 2–5 years' span.^6,11–13^ The stent begins to irritate the femoral-iliac artery once the drug is completely released. The irritation and inflammation is related to poor long-term clinical outcomes. To overcome such issues, development of drug eluting bioresorbable stent (BRS) is required.

A variety of methods for BRS fabrication currently exist, however each are faced with its own limitation. In the case of bioresorbable metal stent, the rapid degradation time may not suffice the time required for the target tissue to be sufficiently healed.^14^ As of biodegradable polymer stents, most are made from poly-l-lactic acid (PLLA), of which the weak material property may lead to stent fracture.^15^ An ideal BRS should be visible during the surgical insertion and maintain both drug release and structural integrity until the tissue of interest is fully recovered.

Yet, compared to metal stents, such type of stent does not possess high radiopacity and therefore are not easily observed under fluoroscopy.^16^ Therefore, observation of BRSs requires an indirect method such as incorporation and tracing of a gold marker, which is still difficult in observing the entire stent structure.

In addition, most stents are loaded with drugs such as paclitaxel and sirolimus in order to prevent restenosis and other poor prognosis.^17^ However, a majority of the loaded drug is released before the stent is degraded in its entirety, making it difficult for the stent to function without causing restenosis.^7^

**Fig. 1:**
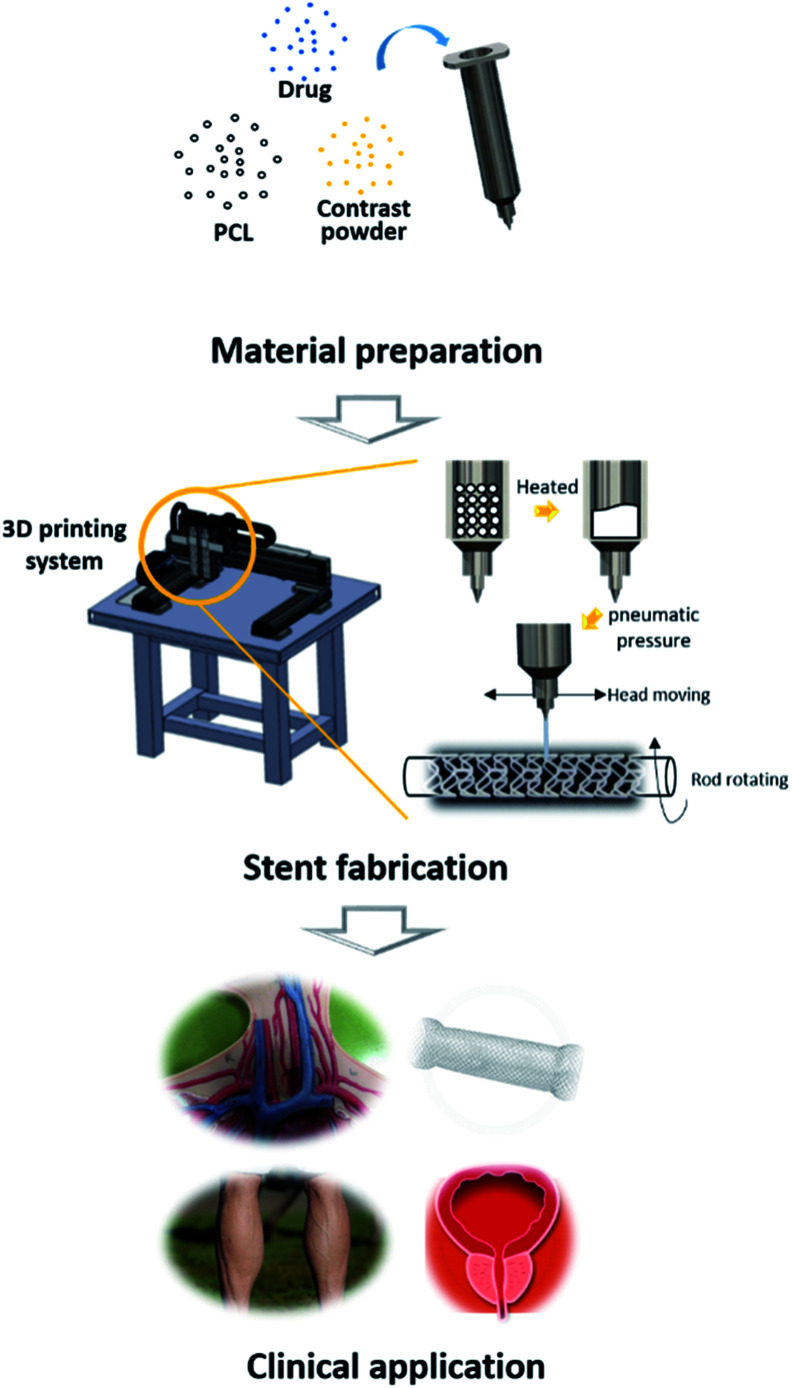
Schematic image of this research. First, the materials used for printing are homogeneously mixed, delivered in a syringe, and mounted on 2RPS. Heat is applied to the syringe to melt the materials inside, and is extruded through the nozzle using pneumatic pressure. At the same time, the syringe moves based on the G-code so that the material can be printed at the desired location. Through this process, various forms of stents can be produced.

In this study, we established a rotating rod combined 3D Printing System (2RPS) to fabricate a cylindrical structure by printing monolayer or multilayer materials on a rotating rod which operates using G-code. The developed system enabled a versatile fabrication of various structures of the target tubular structure. Such fabrication method allows for not only the design but also the functions that can be achieved through the conventional laser cutting method. In this study, a bioresorbable polymer loaded with a drug was printed and the ease and quickness of the method for preparing the stent was confirmed. In addition, a contrast powder loaded BRS was prepared to enable real-time fluoroscopic observation. The developed stents were then safely implanted in the iliac arteries of pigs (Fig. 1).

## Materials and methods

### Materials preparation

Polycaprolactone (PCL; *M*_W_ = 43 000–50 000, PolyScience Inc., Warrington, PA, USA), an FDA approved synthetic bioresorbable polymer, is mainly used for this study as a bioresorbable material. Paclitaxel (Genexol®, Daejeon, Korea) was purchased from Samyang Biopharmaceuticals Corporation. Other chemicals and solvents were purchased from Sigma-Aldrich and used without further purification. Paclitaxel and iohexol were agitated uniformly with molten PCL on a hot plate (110 °C).

### Rotating rod combined 3D printing system (2RPS)

The 2RPS, a one-a-of-a-kind 3D printing system, consists of a driving unit and an extrusion unit. The driving unit is operated by a G-code based robot, which is equipped with two heads moving translationally in the *z*-direction on the moving part of *x* and *y*. The rotating rod for printing the stent is mounted on the rotating axis and moves forward or backward or stays stationary.

The extrusion unit consists of a heater and a dispenser. The heater is mounted on two heads, each of which can heat up to 250 °C to print the material in the syringe. The molten material is extruded by a dispenser capable of providing a pneumatic pressure from 0 to 700 kPa, and the extrusion volume of the material is controlled mainly by the pneumatic pressure and the nozzle size.

There is a rod that can be a substrate for printing the tubular construct. The driving unit and the extrusion unit are controlled by the G-code, and the target material can be printed on the substrate on the plane or rod. Molten PCL was printed on the rotating rod to fabricate the stent, and the strut of the fabricated stent was 350 μm.

For *in vitro* study, paclitaxel and the iohexol loaded stent was used. For *in vivo* study, the stent was loaded with iohexol only for visualization.

### Drug releasing test

Releasing solution contains 1X phosphate-buffered saline (PBS) with 0.05% Tween 80. The solution was placed in a conical tube in with a volume of 10 ml, and shaking was performed at 37 °C in an oven at 10 rpm upon the addition of a sample with bioresorbable stent (diameter: 6 mm, length: 20 mm). 50% of the total solution obtained at each time (days 1, 2, 4, 8, 16, 32, 64, 128, 256, 512) point were analysed by Ultimate 3000 high performance liquid chromatography (HPLC) system (Dionex, Sunnyvale, CA) and refilled with newly prepared solution.

### Stent recoil and radial force

The stent recoil was observed by measuring the outer diameter of the stent 5 days after the ballooning. Radial force was tested with compression test machine (Instron, Norwood, MA, USA). The stent was fixed with a jig for preventing migration during measuring and the tubular shaped probe compressed the middle of the stent with cross-sectional direction for measuring the radial force.

### X-ray imaging

A stent loaded with contrast powder was observed under real-time X-ray using digital angiography system (AXIOM Artis, SIEMENS, Germany) which is widely used for vessel angioplasty. For the *in vitro* imaging, the stent was covered with hand. For *in vivo* imaging, the image was taken during and after stent implantation.

### Animal experiment

A total of 10 stents were implanted in the iliac arteries of 5 pigs with weights in the range of 50–60 kg. Femoral artery was punctured and 8 French, 10 cm length sheath was inserted. 0.035 inch guidewire (Amplatz Super Stiff™, Boston Scientific Inc., Massachusetts, USA) was advanced into the aorta. 6 × 30 mm BRS was crimped on 6 × 40 mm angioplasty balloon. BRS was implanted up to 12 atm for 5 minutes. Peripheral angiography was performed to check deployment of BRS. 300 mg aspirin and 300 mg clopidogrel were loaded before procedure and 100 mg aspirin and 75 mg clopidogrel maintained for 8 weeks. Follow-up was made for 8 weeks after procedure to evaluate adverse events (death, stroke, myocardial infarction, limb ischemia). The pigs were sacrificed after 8 weeks for post-mortem examination. Gross inspection of iliac artery and H&E staining for cross sections of the iliac artery was performed to estimate restenosis and stent apposition.

## Results and discussion

In the conventional 3D printing system, a construct is formed upon the deposition of materials on a flat substrate. Through the layer-by-layer stacking method, various constructs designs of such as ear,^18^ nose,^19^ and breast^20^ are made and are inserted into the patient's body. However, such approach is limited in producing a cylindrical construct due to not only its complicated manufacturing protocol, but also difficulty in yielding a robust structure.^21^ Such aspects of producing a cylindrical construct have been the major barriers for stents to be used in the clinics.^22^ In addition, previous studies on fabricating biodegradable stents based on 3D printing did not: make use of the clinically applicable FDA-approved materials,^23^ result in a high quality shape,^24^ or confirm applicability to native blood vessels.^25^ In addition, no studies on stent exist that maintain a long-term drug release profile along with its radiopaque capability. In this study, a stent is developed containing the features of radiopacity and a long-term drug release profile, while various structural forms can be produced as well.

### Development of rotating rod combined 3D printing system (2RPS) and BRS

**Fig. 2:**
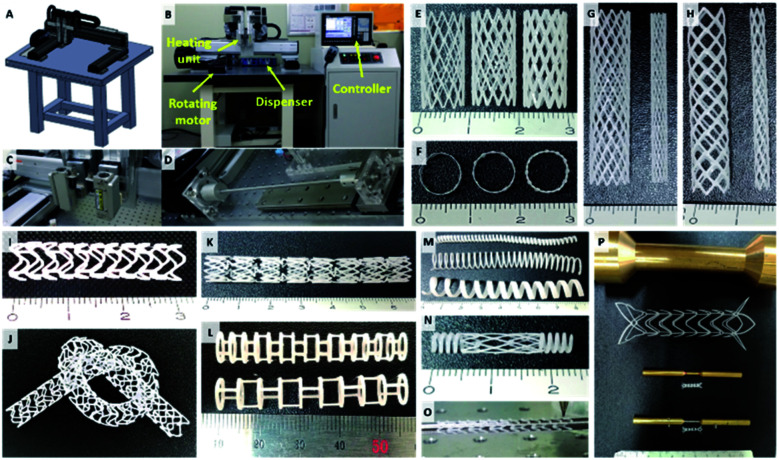
(A) CAD image of Rotating rod combined 3D printing system (2RPS), (B) established whole system of 2RPS, (C) heating unit of 2RPS, (D) rotating unit of 2RPS, (E) various line width of stent (left: 300 μm/mid: 450 μm/right: 600 μm), (F) various wall thickness of stent (left: 200 μm/mid: 300 μm/right: 500 μm), (G) 12-peak stent with different stent diameter (left: 8 mm, right: 3 mm), (H) 6-peak stent with different stent diameter (left: 8 mm, right: 3 mm), (I and J) open cell type stent, (K) closed and open cell hybrid stent, (L) tubular type stent, (M) coil type stent, (N) coil, mesh hybrid stent, (O) protrusion surface imbedded stent for preventing migration, (P) various size of dumbbell type stent.

In this study, we developed 2RPS, which is a 3D printing system for printing materials on cylindrical substrates (Fig. 2A–D). In this system, the movement of the printing head and the rotating rod is controlled by the G-code. The drug loaded polymer is mounted on a syringe, dispensed through a nozzle using pneumatic pressure, and a cylindrical shape is produced by printing the material on the rod. (Video 1†) The strut width, wall thickness, *etc.* of the stent can be changed by adjusting fabrication properties such as feed rate, stacking time, and diameter of rod (Fig. 2E–H). In addition, a stent of the desired shape could be produced for various purposes.

In this study, various types of BRS were developed including: a flexible type stent with a closed cell or an open cell (Fig. 2A–C), a single layer coil, mesh, or combination type stent (Fig. 2D–F), and a protrusion surface imbedded or dumbbell shape stent targeted at preventing stent migration (Fig. 2G and H). Printing of various designs is possible since the 2RPS can perform translational movements along all directions in the three dimensional axes combined with rotational movement from the rotating rod as a printing substrate. This shows that the stent geometry can be changed freely according to the desired purpose. The 2RPS enables the fabrication of stents with multiple designs, which could not be achieved by the conventional laser cutting method.

### Stent recoil and radial force test of BRS

**Fig. 3:**
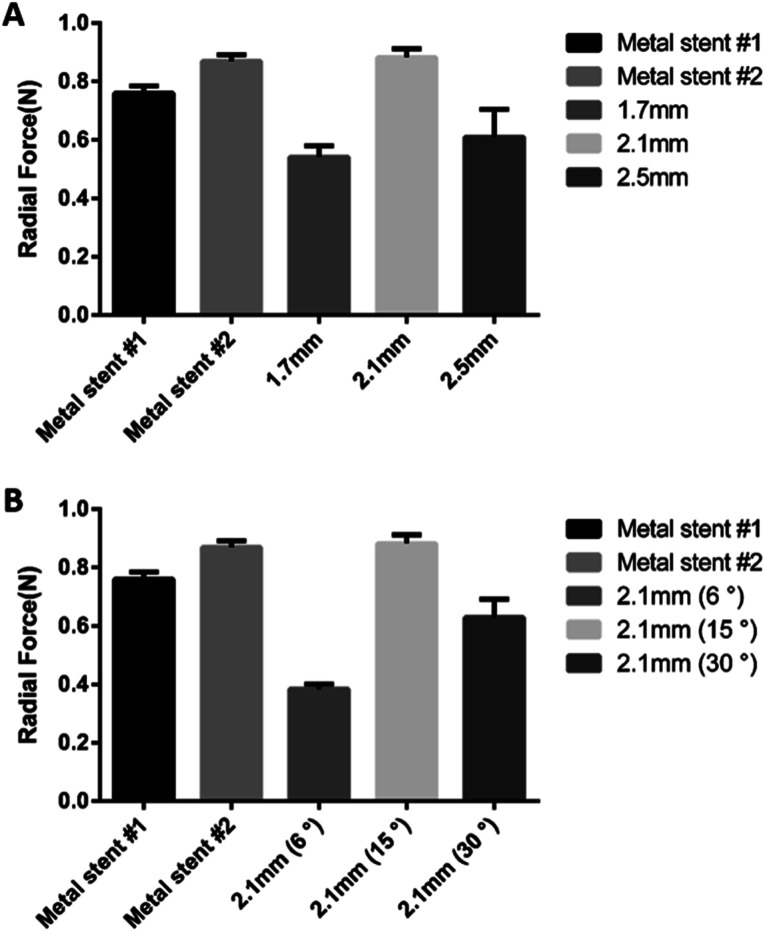
Difference of the radial force of BRSs according to strut length (A) and degree (B).

Stent recoil and radial force indicate the physical properties of the stent, and are the measure of reliability of the stent as a medical device for implantation. It is necessary to fabricate a stent with a radial force similar to that of a conventional metal stent without a stent recoil. Because the BRS is not made from metal, concerns on stent recoil and radial force need to be addressed. We confirmed the change in radial force according to the design of the stent strut. Stents with 2.1 mm pitch length and 15° pitch degree exhibited a radial force similar to the commercial metal stent (Fig. 3). Stent recoil did not occur in the BRS after its deployment (Fig. S1†).

### Drug eluting test of paclitaxel loaded BRS

Stenosis can occur in the tubular tissues in our body from various causes.^26,27^ For such symptoms, a drug-loaded stent is applied. However, the current methods cannot provide enough drug release profile to prevent restenosis.^7^

The conventional metal stent has been reported with a high rate of restenosis in the atherosclerotic femoral-iliac artery disease. The stent in the femoral-iliac artery irritates and inflames the artery continuously. To suppress the inflammation, drugs such as paclitaxel and sirolimus are coated on the surface of the stent. However, the symptoms may reoccur and lead to restenosis after the loaded drugs are completely released, typically within 6 months. In this study, the BRS was made by incorporating paclitaxel in the polymeric material to prevent restenosis while the stent is absorbed in the femoral-iliac artery.

**Fig. 4:**
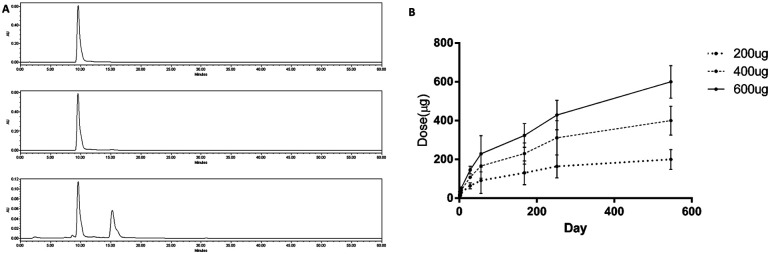
(A) Heat resistance test of paclitaxel. Top: non-heated/middle: 8 h 120 °C heated/bottom: 8 h 200 °C. (B) 1.5 years release profile of paclitaxel from BRS.

In the case of using PCL as a printing material, caution is required in loading the drug as the thermo-sensitive drugs may be damaged due to the heat emitted by the molten PCL during the printing process. It was confirmed that paclitaxel was stable at the printing temperature, and thermal degradation was not observed upon confirmation with HPLC after heating at 120 degrees for 8 hours (Fig. 4A). To confirm the release profile, each stent was loaded with 200, 400, and 600 μg of paclitaxel.

Paclitaxel loaded stent showed a constant release of 1.5 years (Fig. 4B). In the case of PCL, the degradation time is typically known to be 2 years. This means that the paclitaxel will be continuously released until the PCL is degraded, providing sufficient time for the stent to be absorbed and prevent restenosis.

### Visualization of BRS

One of the biggest issues in using bioresorbable stent is visualization. In the case of metal stent, it is easily visualized in peripheral angiography. On the other hand, bioresorbable polymer based stents cannot be detected as the X-rays penetrate the structure, whereby the problem is solved by an indirect visualization method such as attaching a gold marker.^16^ However, it is difficult to confirm that the entire stent is properly implanted. Addressing this issue, stent material was prepared by mixing contrast powder directly in the molten bioresorbable polymer.

Mixing large amounts of powder in the polymer can affect the printability of the materials. The printability was verified by various feedrate by mixing 10 and 20% of iohexol used as a contrast powder. Under constant pneumatic pressure (600 kPa), the line width could be controlled in the range of 50 μm to 550 μm. The line width slightly decreased upon higher content of contrast powder in the PCL mixture (Fig. S2†).

**Fig. 5:**
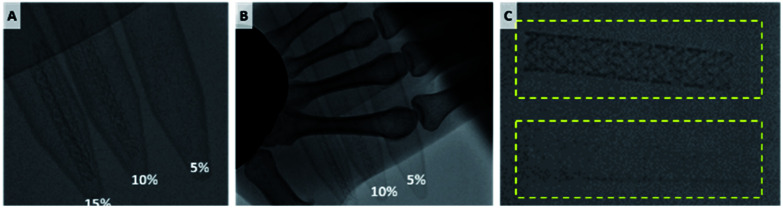
Fluoroscopic image of contrast powder loaded BRS. (A) 5, 10, 15% iohexol loaded BRS. Visibility of the BRS (B) when covered with hand and (C) after washing (bottom).

The stent was loaded with 10% iohexol, a widely used contrast medium for angiography, and was easily visualized under fluoroscopy. When the stent was covered with hand, it was visible under fluoroscopy when it contained 15% iohexol (Fig. 5).

In addition, fluoroscopy was taken after shaking at 10 rpm for two months in saline solution to see if the stent was retained visually after a certain time after insertion into the blood vessel. Even after 2 months of washing, the BRS showed adequate visibility. The view was clear enough for the clinician to recognize the implantation points, stent under expansion, and stent migration without the aid of additional devices.

### Animal experiment of BRS

**Fig. 6:**
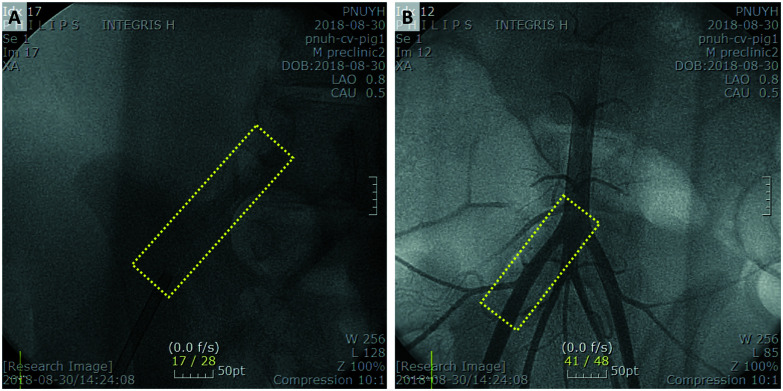
(A) Implanted BRS is visible under fluoroscopy in the right iliac artery of porcine. (B) Peripheral angiography shows that BRS is located in the right iliac artery.

To confirm safe deployment, short term safety, and visibility of the stents, animal experiments were conducted using the BRS. Balloon expandable BRS was prepared to be inserted in the artery. The femoral artery of the pig was punctured and the BRS was implanted in the iliac artery. Ten BRSs were inserted in each iliac artery of 5 pigs. Pigs were sacrificed after 8 weeks for post-mortem examination and confirmed that the BRSs were implanted in the iliac arteries without migration. The BRSs provided sufficient visibility during procedure, as shown in Fig. 6A, allowing accurate stent implantation in the iliac arteries. Peripheral angiography showed that the BRSs were visible in the iliac arteries of pigs (Fig. 6B).

**Fig. 7:**
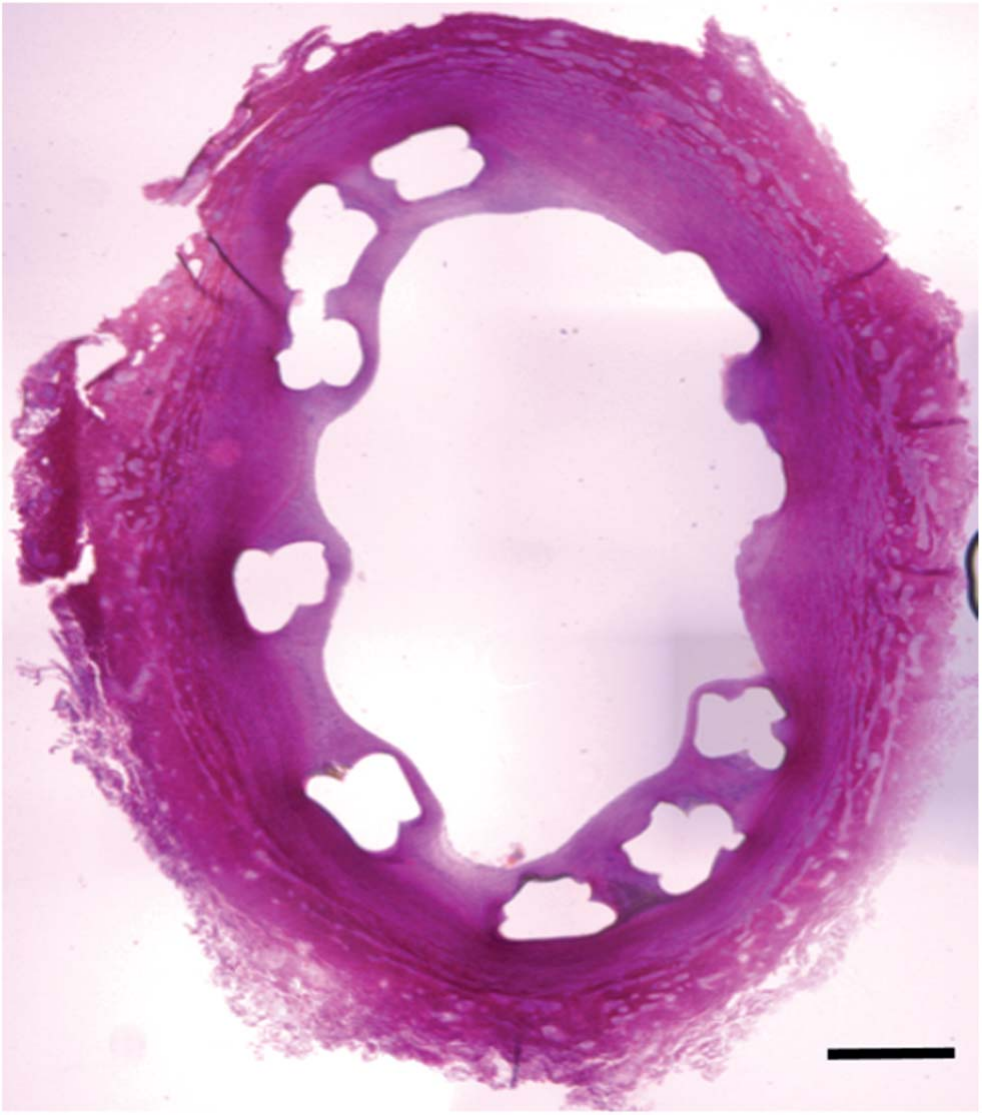
Cross section of the iliac artery show good apposition of BRS. H&E staining confirmed the neointimal proliferation and absence of stent restenosis (Scale bar: 500 μm).

There was no adverse event of death, stroke, myocardial infarction and limb ischemia for 8 weeks after insertion of BRSs (Fig. 7).

## Conclusions

A stent was easy to manufacture using the G-code based 3D printing system in terms of flexibility in changing its design which also enabled the fabrication design that were previously not achieved by the laser cutting method. In addition, a drug eluting BRS was produced which was capable of releasing the drug type specific to the target disease. At the same time, to solve the problem of real-time observation under fluoroscopy, we loaded different types of contrast powder with successful visualization. Furthermore, the BRSs were implanted in the iliac arteries of pigs without any adverse events. In summary, a radiopaque, customized drug eluting BRS was successfully developed. With favourable results shown by the pilot study, a further pre-clinical animal study for long-term pre-clinical study will be conducted. We strongly believe that our innovative research result can be the cornerstone to the development of next generation stents.

## Conflicts of interest

There are no conflicts to declare.

## Supplementary Material

RA-009-C9RA06179G-s001

RA-009-C9RA06179G-s002
