# Supplementary material for: Development of a radiopaque, long-term drug eluting bioresorbable stent for the femoral-iliac artery
Source: RSC Adv. 2019 Oct 28;9(59):34636–41. doi: 10.1039/c9ra06179g (PMC9073948; doi:10.1039/c9ra06179g)
Supplement: RA-009-C9RA06179G-s001 [file RA-009-C9RA06179G-s001.pdf]

Electronic Supplementary Material (ESI) for RSC Advances.
This journal is © The Royal Society of Chemistry 2019

**Electronic Supporting Information for**

# Development of radiopaque, long-term drug eluting bioresorbable stent for femoral-iliac artery

Dong-Heon Ha[a], Jae Yun Kim[b], Tae Sik Park[c], Jong Ha Park[c], Suhun Chae[a], Byoung Soo Kim[a], Han Cheol Lee[c], and Dong-Woo Cho[a]

[1] Department of Mechanical Engineering, Pohang University of Science and Technology (POSTECH), Pohang, Gyeongbuk, Korea

[2] School of Interdisciplinary Bioscience and Bioengineering, Pohang University of Science and Technology (POSTECH), Pohang, Gyeongbuk, Korea

[5]Division of Cardiology, Department of Internal Medicine, College of Medicine, Pusan National University, Busan, Korea

Corresponding E-mail: dwcho@postech.ac.kr

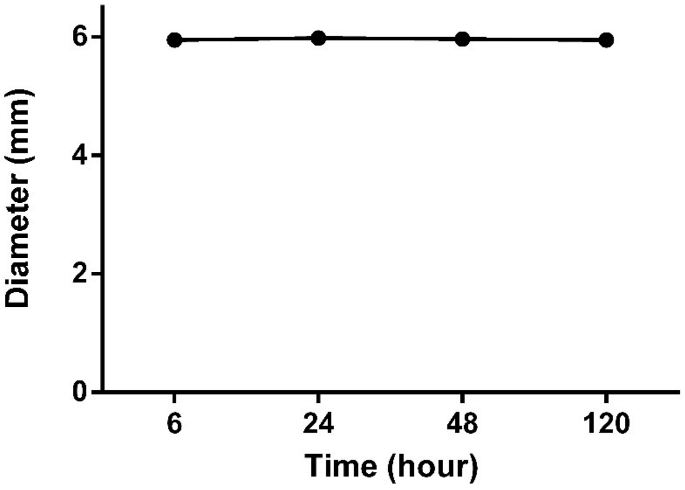


Fig S 1. Stent outer diameter of expanded BRS stent

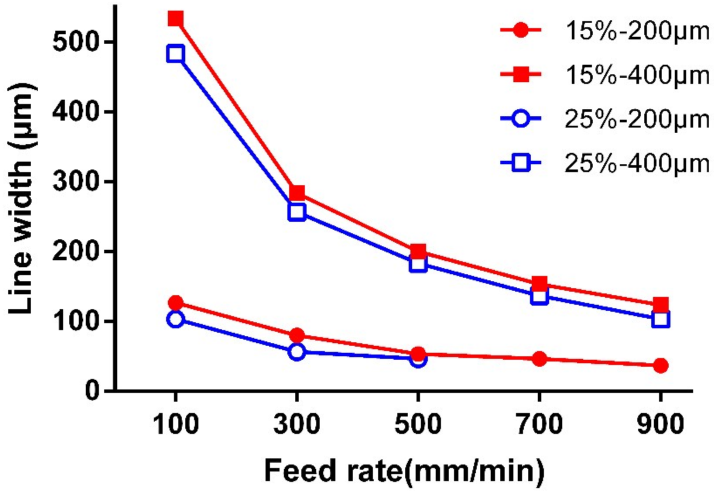


**Fig S 2.** Printing line width of the contrast powder mixed PCL (concentration – nozzle diameter)
